# Long-Term Complications of Aquafilling Injection after Male Breast Augmentation: A Case Report

**DOI:** 10.3390/life14081048

**Published:** 2024-08-22

**Authors:** Anna Fast, Christine Radtke

**Affiliations:** Department of Plastic, Reconstructive and Aesthetic Surgery, Medical University of Vienna, Waehringer Guertel 18-20, 1090 Vienna, Austria

**Keywords:** surgical management, breast deformity, permanent filler, inflammatory process, case report

## Abstract

(1) Background: Aquafilling, a filler initially designed for contouring the face, was developed in 2005 by the company Biomedica, Czech Republic. It is composed of 98% hydrophilic gel and 2% copolyamide. Since 2008 the use was expanded for augmentation of the breast and gluteal region. It is suitable as a nonsurgical method for patients who want discreet augmentation and would like to avoid undergoing an operation. This method promises long-term results lasting up to 10 years. However, according to the literature Aquafilling gel can cause long-term complications; (2) Methods: This case report presents a male patient who experienced severe complications following Aquafilling injections for breast augmentation after ten years of treatment; (3) Results: A 51-year-old male patient presented to our emergency room with complaints of pain, firmness, major pre-pectoral swelling and asymmetry in his breasts. Due to the severity of the clinical appearance and the patient’s discomfort, surgical intervention was deemed necessary. After undergoing three operations the patient was treated successfully and could be discharged for outpatient controls. The patient still did not show any signs of complications, three months after the event; (4) Conclusions: Although the patient has made a rapid recovery, as a result of the surgical procedure, we would recommend treating the breast region with small incisions and drainage in the future. This would have a better aesthetic result. Still there is no antidote available for the treatment of Aquafilling gel. It can only be removed by surgical debridement or irrigation and drainage.

## 1. Introduction

In the past, different methods were established for breast augmentation, such as silicone, paraffine, polyacrylamide gel or fat grafting [1,2]. Aquafilling, a filler initially designed for contouring the face, was developed in 2005 by the company Biomedica, Prague, Czech Republic. In recent years, the injection for augmentation of the breast and gluteal region has risen [3,4]. It is composed of 98% hydrophilic gel and 2% copolyamide [5]. A former non-degradable filler Polyacrylamide gel (PAAG) manufactured in the 1980s, called Aquamid, has a very similar composition and was prohibited after irreversible destruction to the breasts [6,7] This method promises long-term results of lasting up to 10 years, unlike other fillers for example hyaluronic acid, which declared augmentation for 3 up to 12 months, so-called medium-term filler [8,9]. It is an appealing method because it can be used to augment the breast in a minimally invasive way without surgery [10]. No authorization was granted by the US Food and Drug Administration, because of the multiple reported complications [11] It remains controversial in terms of efficacy and safety, considering that there are already some described cases with complications in the literature [3,5,10].

## 2. Case Report

**Figure 1:**
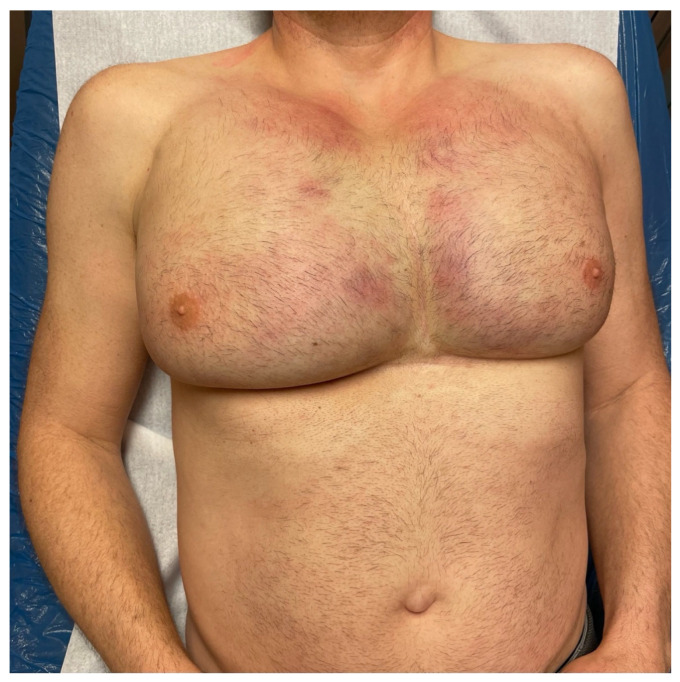
A 51-year-old man received Aquafilling filler injections twice in both breasts, 5 and 10 years ago. The right breast was more swollen than the left breast and showed signs of hematoma.

A 51-year-old male patient presented to our clinic with complaints of pain, firmness, major prepectoral swelling and asymmetry in his breasts. He reported about high temperature of up to 41° degrees at home, in our clinic we measured 37.8 °C. He had undergone breast augmentation with Aquafilling injections 10 years ago in another private clinic in Germany. A touch up followed five years later. His complaints persisted for two days at the time of presentation. He suffered from a respiratory infection for the past five days. On examination, the right breast appeared larger than the left one. At both sides fluctuations were palpable and very painful (Figure 1).

**Figure 2:**
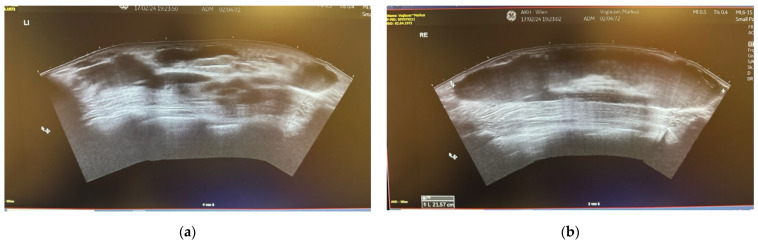
(**a**) Ultrasound of the left breast region; (**b**) Ultrasound of the right breast region.

An ultrasound was conducted to clarify the clinical picture. The imaging revealed on the right pectoral side large subcutaneously located 21.5 × 12 × 5 × 4.5 cm circumscribed fluid formation with hyperechogenic internal foci. Laterally, further smaller circumscribed fluid formations of up to 7.5 cm extending into the axillary extension. The fascia of the pectoralis muscle did not seem damaged, no signs of intramuscular fluid formation was detected. The axillary lymph nodes appeared low reactive. On the left pectoral side multiple accumulations of liquid up to 6 cm revealed. The muscle fascia of the major pectoralis muscle showed interruptions in continuity with adjacent intramuscular fluid formations that extended laterally intramuscularly to the tendomuscular junction. The lymph nodes were also slightly reactive on this side (Figure 2).

As well as the clinical picture the blood sample showed signs of infection, with a leukocytosis of 20 G/L and high CRP of 23 mg/dL. The diagnosis was made clinically and via ultrasound. The clinical examination showed typical signs of inflammation, like rubor, tumor, dolor, calor and functio laesa. The ultrasound showed an extensive fluid formation prepectoral on both sides and on the left side with intramuscular parts with a clear edematous surrounding reaction, primarily valued as superinfection after filler injection.

**Figure 3:**
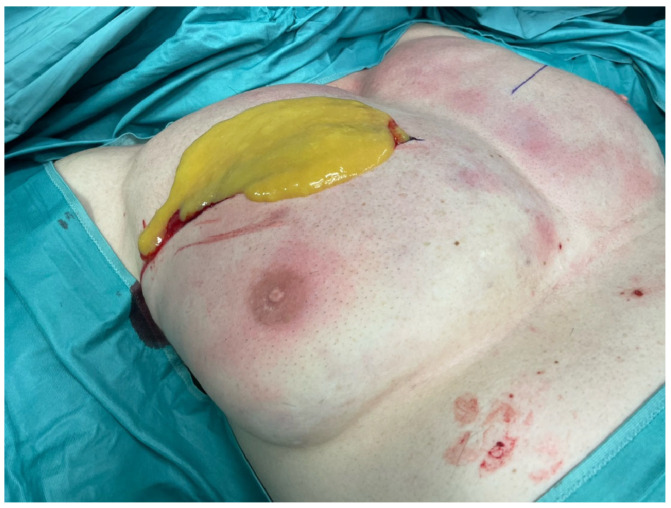
The intraoperative findings in the right breast at the first surgery.

**Figure 4:**
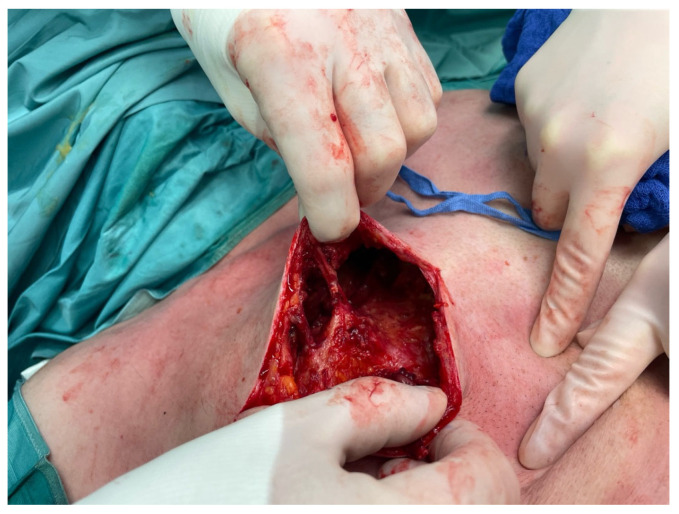
Breast tissue intraoperative in the right breast during debridement.

**Figure 5:**
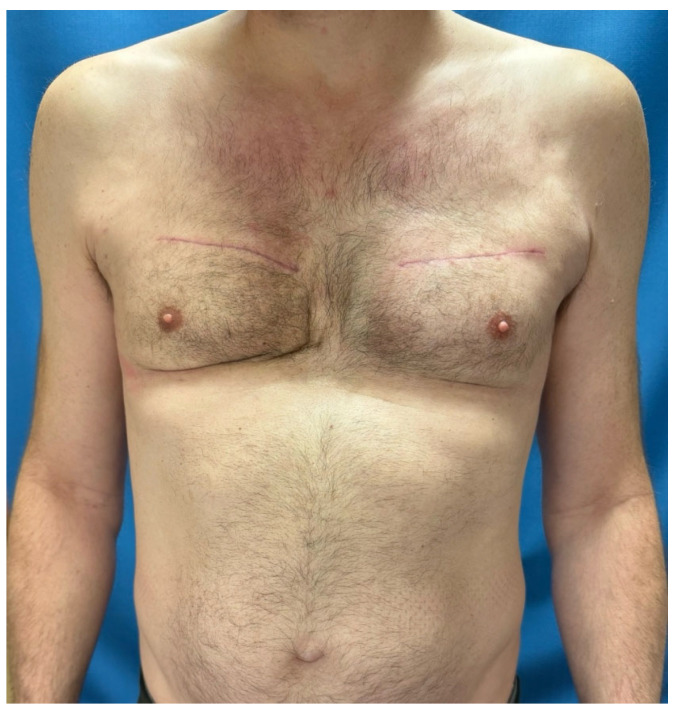
Nine weeks postoperative clinical photograph of follow-up.

Due to the severity of the clinical appearance and the patient’s discomfort, surgical intervention was deemed necessary. Bilateral explantation of Aquafilling material around 700 mL on each breast was performed. Three operations were performed in the course of his hospital stay of 9 days at intervals of 2 days (Figure 3 and Figure 4). In the first operation debridement was performed and necrotic fatty tissue was visible on all sides, also intramuscularly between the fibers of the pectoralis major muscle. The fatty tissue was dissolved completely but the skin was intact and showed no signs of hypoemia. All perforators were not involved in the destructive process. The microbiological smear test showed no growth of bacteria. The histology described inflamed foreign material and inflamed tissue. In the final operation redon drainages were implemented. The postoperative recovery was uneventful, and the patient reported resolution of pain and improved breast symmetry. Under ongoing antibiotic treatment, the signs of inflammation in the blood, and in the local status of the breasts on both sides regressed. Regular follow-up appointments were scheduled to monitor the patient´s progress postoperatively. At nine weeks follow up the patient remained satisfied with the outcome of the surgery, with no recurrence of complications (Figure 5).

## 3. Discussion

The use of less invasive methods, such as injectable fillers, for body contouring and augmentation is popular. Aquafilling is a dermal filler applied to the face to improve small corrections. It was approved for breast augmentation in Europe in 2008 [5]. Aquafilling offers several advantages as a facial rejuvenation technique, including its minimally invasive nature and long-lasting results. The disadvantages described in multiple publications are the inflammatory reactions, some become prominent years after injection, the problem of migration of the filler and lumps and deformities of the treated area [3]. Aquafilling injections for breast augmentation can lead to various complications, including asymmetry, capsular contracture, and migration of filler material. Hyaluronic acid fillers are applied in smaller amounts in the face. It can last up to 12 months, which is a much shorter period of time, as Aquafilling gel. If complications arise, hyaluronic acid can be dissolved with its antidote hyaluronidase [10]. There is no antidote for Aquafilling filler, so surgical intervention, such as explantation may be necessary to address these complications effectively. Patients should be informed about the potential risks associated with Aquafilling gel and encouraged to consider FDA-approved alternatives for breast augmentation [10]. A greater filler volume appears to raise the chances of complications occurring [12].

A study published of Shin et al. reported long-term results of additional filler injected after breast augmentation with silicone implants for correction of small imperfections after the surgical procedure. They described two women with follow up time of 1, 3 and 6 months after treatment. The outcome was satisfying without any complications reported. The limitation here is the short period of time of the follow up councils [13]. The literature mainly describes case reports of female patients. One case report of a male patient is described for penis augmentation with Aquafilling, which also states major long-term complications. This was also accompanied by severe infection which, however, also destroyed the skin mantle. As a result, reconstruction by means of flap surgery was necessary [14]. Our case is a male patient who underwent breast augmentation with Aquafilling gel. A comparable case in a male patient has not yet been described in the literature. The long-term complications observed with Aquafilling filler are concerning and can occur years after the initial procedure. Infections can occur due to contamination during the procedure. The formation of granulomas is a significant delayed complication. Also described in the literature are filler migration and displacement [15]. This can lead to asymmetry or displacement into unintended areas, causing concerns that might require corrective surgery [3].

Our patient showed inflammatory signs of the breast for 2 days, after having Aquafilling filler as augmentation of the breasts 5 and 10 years ago at a private practice. The patient reported about having a respiratory infection 5 days ago. Until then he did not notice any symptoms in his breast region. One case of an autoimmune reaction to the Aquafilling filler after COVID-19 infection is described in the literature [16]. Our patient was not screened for a COVID-19 infection. We would like to express a possible connection of a cross reaction to the Aquafilling gel after a respiratory infection.

The histology described an inflamed tissue and foreign body reaction as well as necrotic tissue. During the operations all the perforators did not seem involved in the destruction process. In our case, the entire fatty tissue appeared dissolved. There was only a vital skin mantle, glandular tissue and muscle. A study reports about histological changes of the tissue after Aquafilling gel. Chalcarz et al. describes numerous immune system cells involved in the inflammatory process [17].

In this case report, the incision line was defined by the surgeon because of the massive extent of the inflammation. This enabled access to the anatomical limits. The ultrasound evaluation showed the extend from the parasternal line to the front axillary line up to the clavicula and caudally to the submammary fold. The literature only describes cases of complications after Aquafilling injections in women´s breasts. In breast surgery of the female breast, it is typical to make the incision line in the submammary fold [10,18,19]. Further case reports show sufficient treatment by small incision and drainage in the inframammary fold, followed by lavage or irrigation. If necessary, the incision in the inframammary fold can also be widened, as in the case of augmentation, to facilitate exploration. As in our case, several operations are necessary [4,5,10].

This case report highlights the potential risk of long-term complications after Aquafilling injections. In addition to existing studies, this case describes a further long-term complication of Aquafilling filler. Based on our experience and data of the literature we would recommend refraining from an injection with Aquafilling gel [5,10]. In future cases, it is certainly advisable to carry out a histological examination of the tissue. Further research is important because it is still unclear which possible consequences may still occur after the treatment with Aquafilling filler. Long-term studies are warranted to further evaluate the durability and safety profile of Aquafilling. This case highlights potential complications associated with Aquafilling injections and underscores the importance of thorough preinterventional evaluation and informed consent. Surgeons should exercise caution when considering the use of Aquafilling and prioritize patient safety. In our case report, no re-inflammatory process was yet observed.

## 4. Conclusions

To summarize, Aquafilling has been in use since 2005. It has shown satisfactory results for breast augmentation, facial and body augmentation, but also numerous long-term complications. Thus, the long-term safety and effectiveness cannot be observed [10,11]. In our case report, the patient showed high signs of inflammation, feeling sick and pain. It is not clear whether this case was caused by a cross reaction of the body due to a previous respiratory infection. In view of these observations, the Aquafilling filler should not be used for breast augmentation or any other application in humans until its safety has been verified. It is advisable to use a filler with an existing antidote. Furthermore, established methods such as lipofilling or silicone implants for men should be used to achieve a satisfactory result with a lower complication rate [20,21]. This case report was a precedent at our clinic. Even though we acted this way in our case, we had a learning curve with this case and would in future treat male and also female via mini-incisions and drainage in the inframammary fold, in several sessions. This shows another case report describing severe long-term complications following Aquafilling gel injection. Still, there is no antidote available for the treatment of Aquafilling gel. It can only be removed by surgical debridement or irrigation and drainage. The first approach in cases like this should always be drainage over mini incisions.

## Data Availability

The data presented in this study are available on request from the first author. The data are not publicly available due to policy restrictions.
